# New Values, New Lives, and Emerging Dating Violence: Insights on Detection and Intervention from Health Sciences Students

**DOI:** 10.3390/bs16050630

**Published:** 2026-04-23

**Authors:** Sara Sanchez-Balcells, Maria Aurelia Sánchez-Ortega, Marta Prats-Arimon, Pepita Giménez-Bonafé, Núria Vergés Bosch, Montserrat Puig-Llobet

**Affiliations:** 1 Departament d’Infermeria de Salut Pública, Salut Mental i Maternoinfantil, Facultat d’Infermeria, Universitat de Barcelona, 08907 L’Hospitalet de Llobregat, Barcelona, Spain; sara.sanchez@ub.edu (S.S.-B.); monpuigllob@ub.edu (M.P.-L.); 2 Research Group in Mental Health, Psychosocial and Complex Nursing Care (NURSEARCH), Facultat d’Infermeria, Universitat de Barcelona, 08907 L’Hospitalet de Llobregat, Barcelona, Spain; 3 Departament de Fonaments Professionals i Gestió en Salut, Facultat de Ciències de la Salut i el Benestar, Universitat de Vic, 08500 Vic, Barcelona, Spain; mariaaurelia.sanchez@uvic.cat; 4 Departament d’Infermeria Fonamental i Clínica, Facultat d’Infermeria, Universitat de Barcelona, 08907 L’Hospitalet de Llobregat, Barcelona, Spain; 5 Departament de Ciències Fisiològiques, Facultat de Medicina i Ciències de la Salut, Campus Bellvitge, Universitat de Barcelona, 08907 L’Hospitalet de Llobregat, Barcelona, Spain; pgimenez@ub.edu; 6 School of Sociology, Universitat de Barcelona, 08907 L’Hospitalet de Llobregat, Barcelona, Spain; nuria.verges@ub.edu

**Keywords:** dating violence, health sciences university students, psychological violence, aesthetic pressure, interpretative phenomenological analysis, prevention, mental health

## Abstract

Gender-based violence in dating relationships is a multifaceted issue that encompasses diverse forms. In university settings, high prevalence rates have been reported, with psychological violence being the most common. New forms of digital violence, such as cyberbullying, control through social media, and digital aesthetic pressure, further complicate the phenomenon. Purpose: This study aimed to explore Health Sciences students’ perceptions of gender-based violence in dating relationships to identify key dimensions for understanding and intervention. Methods: A qualitative design was employed using focus groups with ten participants, analyzed through Interpretative Phenomenological Analysis (IPA). Results: Four main themes emerged: characteristics of gender-based violence in dating relationships, types of violence identified, aesthetic pressure within affective relationships, and strategies for detecting and responding to violence. Conclusions: Findings emphasize the importance of incorporating students’ voices into prevention strategies and propose educational interventions that address both offline and online dynamics of gender-based violence in dating relationships.

## 1. Introduction

Gender-based violence in dating relationships constitutes a multifaceted problem that encompasses various forms, including physical, emotional, verbal, social, and sexual forms, within the context of affective relationships during both adolescence and adulthood (Darojat et al., 2023; Lewis & Fremouw, 2001). This type of violence may manifest unilaterally or reciprocally, and its expression is influenced by individual, relational, and sociocultural factors (Courtain & Glowacz, 2021). In recent years, the development of digital technologies has introduced new forms and domains of violence facilitated by electronic means, particularly within relationships initiated or maintained through online dating platforms, thereby broadening the spectrum of risks associated with victimization (Filice et al., 2022).

Prevalence rates of dating violence vary significantly across studies, regions, and population groups, making direct comparison of results difficult (Courtain & Glowacz, 2021; Tomaszewska & Schuster, 2021). Likewise, in university settings, high prevalence rates of dating violence have been reported, with psychological violence being the most common form (Pérez Vázquez & Rubio Guzmán, 2025). In this regard, new manifestations of violence have been observed in digital environments, such as sexual cyberharassment or non-consensual digital pornography (Bosch & Gil-Juarez, 2021) on social media. It often begins gradually and progressively, with psychologically subtle forms of aggression that are difficult to identify, such as humiliation, isolation, hostile behaviors, or attempts to exercise control and power over one’s partner. Its continuation depends on the presence of risk factors such as exposure to physical aggression or psychological violence (Rubio-Garay et al., 2015). Cultural, gender, and age differences influence the experience, perception, and reporting of these episodes, posing methodological and ethical challenges for research (Tomaszewska & Schuster, 2021).

With regard to attitudes and perceptions, it has been observed that some students adopt tolerant or ambivalent stances toward dating violence, even going so far as to justify or fail to identify certain abusive behaviors, such as jealousy or controlling actions, as manifestations of violence (Bilgiç et al., 2023; Freijomil-Vázquez et al., 2022). According to Freijomil-Vázquez et al. (2022), sexist attitudes and traditional gender perceptions are associated with greater acceptance and perpetration of violence, particularly among men. Students’ ability to identify situations of dating violence tends to be limited, highlighting the need to strengthen education and training in this area (Bilgiç et al., 2023).

Among individual risk factors, the most notable are alcohol consumption, prior exposure to family or interparental violence, low self-esteem, and the normalization of violent behaviors (Cohen et al., 2018). At the relational level, the intensity of the bond, jealousy, and peer pressure can act as catalysts for aggressive behavior. At the social level, cultural and gender norms, gender stereotypes, and the use of digital media contribute to the reproduction of violent dynamics, particularly in contexts where adult supervision is limited or absent (Filice et al., 2022).

The consequences of dating violence are multiple and affect both the mental health and the social and academic well-being of students (Courtain & Glowacz, 2021). Short-term and long-term negative effects have been documented, such as anxiety, depression, suicidal ideation, substance use, and poor academic performance, with variations in intensity and duration depending on gender and age (Pérez Vázquez & Rubio Guzmán, 2025). Likewise, according to Courtain and Glowacz (2021), attitudes toward violence and the definitions that young people attribute to these behaviors directly influence their tolerance, the likelihood of reporting incidents, and the effectiveness of interventions.

Taken together, gender-based violence in dating relationships represents a prevalent and well-documented phenomenon among Health Sciences students, with significant implications for both their personal well-being and their future professional practice in the healthcare field. As highlighted in previous research, health professionals play a crucial role in the prevention, detection, care, and recovery of gender-based violence (Alonso et al., 2024). However, the normalisation of certain abusive behaviours, the limited ability to identify non-physical and subtle forms of violence, and the lack of specific training in this area underscore the need for comprehensive educational and preventive strategies. Such strategies should address gender-related attitudes, strengthen recognition of invisible and emerging forms of violence, and reinforce institutional support systems to better prepare future professionals.

Within this framework, the study aimed to contribute to the identification of gender-based violence occurring in dating relationships, as well as to provide Health Sciences university students with tools and strategies to promote the eradication of gender-based violence.

## 2. Materials and Methods

A qualitative study was conducted using an Interpretative Phenomenological Analysis (IPA)–informed approach, following the guidelines proposed by authors such as Smith and Fieldsend (2021). Although IPA is traditionally applied to individual interviews, our study adopts its phenomenological principles within a focus group setting, drawing on the work of authors who support the use of IPA-informed analysis in group-based research (Love et al., 2020). This adaptation allows for the exploration of shared meaning-making and collective sense-construction as they emerged through participants’ interaction.

### 2.1. Recruitment and Participants

The study is based on data collected through focus groups designed to explore participants’ experiences, beliefs, and attitudes through guided interaction (Hayward et al., 2004). Unlike individual interviews, in this format the researcher assumes the role of moderator, facilitating dialogue without directly intervening. This position allows complex group dynamics to emerge, fostering the collective construction of meaning (Bloor et al., 2001). In this study, the focus group was moderated by a female researcher, assisted by another female member of the team, a factor that may also have influenced the interactional dynamics.

This study forms part of a larger multicentre project aimed at improving the detection of dating violence among university students in the Health Sciences. The project involves several universities across Spain and Latin America and uses validated instruments, quantitative assessments and educational innovation activities, all of which are currently in different stages of analysis and publication. Within this broader framework, the focus groups reported in this article constituted the exploratory qualitative component of the project, designed to capture students’ initial perceptions, beliefs and meaning-making processes regarding dating violence. Alongside these focus groups, other methodologies such as media-creation activities—primarily podcast production—were also implemented as part of the educational innovation strand of the multicentre study.

Participants were recruited solely because they were Health Sciences students, and not due to any personal experience of dating violence. Recruitment and participation in the focus groups took place outside regular class hours to avoid any perception of coercion and to ensure that participation was fully voluntary.

The study participants were recruited through a public university located in the metropolitan area of Barcelona, to which the research team also belonged. Several members of the team participated in conducting the focus group, which took place during class hours and was moderated in both Catalan and Spanish. The session was held in a private setting, without the presence of individuals external to the process, thereby ensuring participants’ confidentiality and well-being.

A total of 10 students were selected to participate in the focus group. A detailed description of each participant is provided in Table 1. The sample was selected by convenience and was determined by the Faculty of Medicine or Nursing to which the students belonged. Recruitment was carried out through faculty members, who facilitated contact with the research team. Subsequently, students were asked whether they wished to participate in the groups. Ultimately, two students were excluded for not having signed the informed consent form, and one additional student did not attend the focus group session.

Participants’ ages ranged from 19 to 27 years. Seven identified as female and three as male. They were also asked about their degree program: six were first-year nursing students, and four were second-year medical students. Table 1 presents the participants’ profiles.

The project received approval from the Ethics Committee of the University of Barcelona (Institutional Review Board IRB00003099), under reference code CER062407. All data that could identify participants were anonymized to ensure their privacy. Likewise, all participants signed informed consent forms prior to taking part in the study.

The focus groups were structured around a thematic guide; however, the researchers maintained flexibility to adapt to the participants’ spontaneous narratives. Table 2 illustrates the main themes along with their corresponding descriptions. Table 2 presents the research topics and their corresponding theme descriptions.

No pilot test was conducted prior to the study. The session lasted approximately 90 to 180 min. Recordings were made using a mobile phone and subsequently transcribed with the programs Softcatalà and Whisper (Boté-Vericad & Lopezosa, 2024). These tools were chosen because they support transcription in Spanish, are cost-effective, and meet high privacy standards. Segments that were not recognized by the online tool were added manually, and any detected errors were corrected. In cases of ambiguity or difficulty in comprehension by the primary researcher, a second person was consulted, who had access only to individual statements and did not view the complete transcripts or any personal data. The same team member who moderated the focus group was responsible for reviewing the transcription and correcting potential inconsistencies. In addition, complementary field notes were taken. Repetition of the focus group was not required, as the findings reflected thematic sufficiency within the first session.

Participants were informed about the objectives of the study; however, the potential biases or personal positions of the research team were not explicitly disclosed to them. Three of the ten participants were already acquainted with some of the researchers: one was a faculty member teaching the first-year course in Psychosocial Nursing, while the other two were aware that the team belonged to a university conducting a study on dating violence.

### 2.2. Analytical Strategy

The data collected were examined using the Interpretative Phenomenological Analysis (IPA) approach proposed by Smith and Fieldsend (2021). An abductive thematic analysis approach was applied to identify patterns within the data through a reflexive process aimed at constructing theoretical explanations based on the themes that emerged (Braun & Clarke, 2021; Thompson, 2022). Accordingly, we reframed the analytical description to more accurately present the study as a reflexive thematic analysis informed by phenomenological principles, supported by abductive reasoning to interpret how meanings were constructed within the group context. The analytical procedure was carried out in four phases. In the first phase, a detailed reading of the transcripts was conducted in order to understand both the participants’ responses and their spontaneous reflections, which had been previously stored on the server of the qualitative analysis software Atlas.ti version 25 (Boté Vericad, 2023). In the second phase, relevant excerpts were coded using a deductive approach focused on the research questions concerning dating violence. During the coding process, unexpected narratives emerged related to digital violence and the invisible forms of dating violence among young people, as well as their connection to psychological well-being or distress. These themes emerged in part as a result of the prior research interests of the team members.

Subsequently, an inductive review of the excerpts was conducted to identify themes and subthemes that reflected key concepts, as well as similarities and differences in the attitudes and opinions expressed. Finally, the themes and subthemes were defined and labeled with the aim of faithfully representing participants’ perceptions regarding the detection and management of dating violence. Some excerpts were lightly edited to improve clarity without altering their original content.

Participants were later shown their transcripts in case any corrections were necessary.

This study follows the principles established in the Consolidated Criteria for Reporting Qualitative Research (COREQ) checklist for the reporting of qualitative results (Tong et al., 2007) (see Supplementary Materials).

## 3. Results

This section presents the results obtained. Table 3 summarizes the main categories identified, along with their respective subcategories. The following subsections develop these results in greater detail, focusing on the findings most relevant to addressing the study’s objectives. Table 3 provides an overview of the main categories and subcategories, ranked according to their frequency.

### 3.1. Characteristics of Dating Violence

#### 3.1.1. Expectation in a Relationship

The students analyzed the most significant manifestations of gender-based violence, highlighting a direct relationship with expectations within the couple, especially in the early stages of the relationship. During this period, they reported feeling influenced by idealization and romanticism, as well as by mutual expectations, both in heterosexual and homosexual relationships. Participants described how these expectations affect their perception and evaluation of the relationship, directly influencing satisfaction and intensifying relational conflicts. As one participant (P10) expressed:

“People our age, what I’ve noticed, is that in a relationship we tend to idealize the other person at the beginning and think that they’ll be the kind of person we want them to be. And when we realize they’re not, we experience frustration, often leading to conflict.”

(P10)

#### 3.1.2. Romantic Relationships

The discussions also highlighted how certain possessive or controlling behaviors are frequently romanticized, especially among young people. These attitudes, far from being questioned, are interpreted as expressions of love or commitment, which contribute to their normalization. One participant expressed it as follows (P5):

“And what’s more, people romanticize it. Like, oh, I love you so much that I won’t let you sleep with anyone else. But, you know, you can at least talk to your friends, right?”

(P5)

#### 3.1.3. Types of Violence: Insidious Onset

The accounts revealed how certain dynamics of dating violence may begin subtly and progressively, making them difficult to identify in the early stages. This insidious nature means that, by the time a person becomes aware of the situation, they are often already deeply emotionally involved or socially isolated, which hinders their ability to respond or to leave the relationship. As one participant expressed:

“These kinds of behaviors, by the time you realize what’s happening, you’re already deeply involved and don’t know how to get out, or it’s really hard to do so. You want to leave, but it’s very difficult, and you have no one to turn to.”

(P2)

#### 3.1.4. Student Context

In this regard, participants indicated that the university context, especially when combined with work responsibilities, creates a perceived limitation of time that directly affects interpersonal dynamics. Time management among female students emerged as a source of tension, particularly when those in their social environment do not share the same academic or professional demands.

One participant expressed this clearly (P1):

“Sometimes it’s difficult to balance university and work life with personal relationships, especially when the other person doesn’t share the same pace or level of commitment. I’ve found myself constantly having to justify why I don’t have time, and that can lead to misunderstandings or tension. Not everyone understands what it means to be so organized when they’re not living that same reality.”

(P1)

#### 3.1.5. Relational and Social Conflict

A reconfiguration of significant relationships also emerged, transcending the traditional model of an exclusive, couple-centered bond. This broader perspective allows for the recognition of the importance of multiple affective and social relationships that coexist and intertwine in everyday life.

“We’ve realized that we can have more than one meaningful relationship, not just with one person you love, but with several.”

(P1)

#### 3.1.6. Perception of Violence

A concern emerged regarding the trivialization of violence through humor, particularly in digital environments. Participants pointed out how certain degrading comments or attitudes toward one’s partner are presented humorously, which contributes to their normalization and reproduction, especially among younger individuals:

“…they were joking about something to do with couples, and it’s like, you’re really not helping, you know? Because, as we were saying, a lot of young people nowadays see this kind of behavior and these comments on social media, and they take them in, repeat them, and end up not moving forward with society.”

(P3)

#### 3.1.7. Subtle Violence

One of the most recurrent findings was the difficulty in identifying and naming certain forms of violence that, although not explicit, cause discomfort and affect the university experience (P7, P10, P2, P5).

“I think there are many forms of violence that aren’t visible, that aren’t so obvious, but they’re there and they hurt a lot. Sometimes the most painful thing is not realizing that what you experienced was violence, and then you don’t allow yourself to feel bad or to acknowledge that there was a lack of respect.”

(P7)

These subtle forms of violence are manifested through attitudes of devaluation, silencing, or questioning of intellectual ability, particularly toward women:

“I studied at university ten years ago, and now I’m attending for the second time. In both experiences, I’ve encountered situations of violence, mostly very subtle forms that can’t always be clearly pointed out. It’s often about attitudes like belittling, not listening, or making you feel that you’re not so intelligent. As a woman, I often feel that I’m not taken seriously or that what I have to say isn’t valued.”

(P7)

One of the participants noted how certain messages and attitudes are so deeply embedded in university social life that they go unnoticed:

“I think we also receive a lot of subliminal messages. For example, songs that you might not usually listen to, but that play at parties because they’re trendy. I think there are still many very subtle forms of violence that are present and much more normalized than we think. Sometimes they don’t say anything to you when you go out partying, but the next day they give you the silent treatment. Things like that are also forms of violence, but we’ve internalized them so much.”

(P2)

#### 3.1.8. Racism and Discrimination

Leisure spaces emerge as settings where sexist, racist, and LGBTQ-phobic forms of violence are reproduced, often disguised under discourses of humor or freedom of expression. This normalization makes their identification and questioning particularly difficult, especially among young people.

These discussions also suggest that such forms of violence occur in contexts where differences in values or traditions exist, which can lead to situations of discrimination:

“There are also other kinds of people with more traditional values, or values different from ours, and that’s exactly when these situations happen.”

(P1)

“Obviously, I can joke around with my friends, but I would never do it with someone I don’t know. All these sexist, LGBTQ-phobic, and racist attitudes are often disguised as humor. And many young people who see this on social media take it in and end up repeating those behaviors.”

(P8)

### 3.2. Types of Violence

In this section, it became evident that psychological violence constitutes the most prevalent form of aggression compared to other types. Participants provided concepts that illustrate this form of violence, such as control exerted by one’s partner, moral degradation through derogatory comments, emotional pain, manipulative behavior, indifference or lack of consideration, disrespect, and the absence of intimacy and individuality (P5, P6, P7, P8, P10).

“Every time we met up with her, I could tell she didn’t feel comfortable, as if she were doing something wrong just by being with us. She herself said that her relationship wasn’t going well and that she couldn’t go on with that person…”

(P10)

“You have to kiss me because I’m your partner, or we have to have sex because we’re sleeping together tonight and I’m your partner.”

(P8)

Students also reflected on the imbalance that can arise between a romantic relationship and other aspects of personal life, such as friendship, family, or self-esteem. According to their observations, this imbalance may intensify when the partner becomes the exclusive focus of attention and affection, fostering dynamics of control and emotional dependence. As one participant (P8) expressed:

“Many times, we fail to balance our personal life with our romantic relationship. When we prioritize our partner too much, everything starts to revolve around them, and that can create control, toxicity, and power Dynamics”. Phrases like “Why do you want to go to that place without me?’ reflect that dynamic. When the relationship ends, the person is left very unstable because they had invested everything in it. And many times, we don’t realize this until after the breakup.”

(P8)

“…they shared their location with each other, and whenever she left work, if she didn’t get home at exactly the expected time, it was immediately like: ‘Where are you? Who are you with?’…”

(P1)

Regarding digital violence, participants shared narratives that revealed a constant violation of trust, control exerted through digital means, and the influence of social media in shaping violent relationship dynamics. In many cases, this information was perceived as subtle and normalized, carrying a high risk of being reproduced among younger populations:

“…The fact that these videos appear on social media also makes someone watching TikTok say, ‘Oh, look, it happens to this person too, their partner is also cheating on them.’ That contributes to normalizing it a bit, and if it’s happening to me too, it doesn’t seem as serious as it really is.”

(P4)

“…the cellphone is something private; invading it is a way of violating your privacy, and I see that as a lack of trust…”

(P10)

Other forms of violence also emerged, such as verbal violence, manifested through the imposition of one partner’s desires over the other, as well as physical and sexual violence. Although sometimes less visible, these forms of aggression were recognized by participants as part of relational dynamics characterized by control, inequality, and the violation of consent (P8, P7, P10).

### 3.3. Aesthetic Pressure in Dating

This theme emerged spontaneously in the focus groups and is included in the results not because it was addressed in the research questions, but because it arose directly from group dynamics and participants’ own voices. The topic was organized into four main thematic blocks: first, the importance of social comparison among young people; second, the control of physical appearance influenced by digital media; third, the aesthetic expectations expressed by students; and finally, aesthetic violence understood as a form of systematic pressure and self-evaluation aimed at conforming to normative gender beauty ideals.

Regarding social comparison and aesthetic expectations, participants described how constant exposure to idealized bodies in digital environments affects the way they perceive themselves and their partners. The involuntary comparison with these bodies generates insecurity and self-doubt about one’s own attractiveness and desirability within the relationship.

“There are lots of people and many different bodies on the internet. Of course, that becomes a personal problem. So, you have to start telling yourself, ‘Okay, there are many types of bodies,’ but you can’t help it, you can’t avoid feeling like you do not have the kind of body your partner is looking for, or that you could look better.”

(P6)

In the same vein, narratives emerged that focused on the control of appearance and the subliminal messages received from one’s partner. Participants recounted experiences in which aesthetic pressure did not manifest directly but rather through gestures or implicit messages that generated insecurity. Exposure to idealized body images, even within intimate contexts such as a romantic relationship, can activate mechanisms of comparison and pressure.

“…I get a video of a girl trying on her clothes, and you might start to think that he’s sending it to you, so you’ll see how she looks, and that he’d like you to look like her, right? And yes, that’s a problem, because he’s not saying it to you directly. It’s not exactly a problem he’s causing, so to speak, but I do think there could be more communication on both sides…”

(P9).

In this regard, aesthetic violence emerges from a position that imposes normative bodily standards, particularly on women, shaping their self-perception and interpersonal relationships. In participants’ accounts, this form of violence is not always explicit; rather, it appears in moments of conflict as a symbolic resource used to devalue or control (P5, P10).

“Sometimes, during an argument, things come up that weren’t supposed to be a problem, like whether I remove body hair or not. At first, he didn’t care, but later, in the middle of a fight, he’d say things like I’m disgusting for not shaving. And that makes you think about how deeply ingrained certain beauty standards are, especially for women. Even if they’re not always said outright, that pressure is still there.”

(P4)

### 3.4. Addressing Dating Violence: Detection and Response Strategies

Students’ narratives were organized into a broad thematic block from which various topics emerged, including acceptance and empathy, boundary setting, emotional support, intervention and assistance, self-awareness, assertive communication, relational awareness, inclusion and diversity, educational and preventive measures, critical thinking, and gender equality (P2, P7, P9, P12, P15).

One of the most prominent themes was education in personal values as a key strategy for identifying forms of violence that often go unnoticed in affective relationships. This approach is linked to the ability to recognize subtle signs of emotional distress and to establish boundaries from a position of self-awareness.

“Perhaps, on an individual level, in order to detect those forms of violence that often go unnoticed, it would be necessary to educate people in values. […] If from the start I’m clear that something will make me feel bad in the long term, I can set a boundary and prevent it from affecting me directly.”

(P9)

Closely related to the above, the importance of self-knowledge also emerged strongly as a starting point for establishing personal boundaries. This process is presented as a prerequisite for identifying situations of violence, particularly those that do not manifest explicitly.

“I would say it’s even more important now because, as they say, if you don’t love yourself, you can’t love someone else. It’s about knowing yourself: What are the boundaries you set for yourself? […] By knowing yourself, you can tell whether someone is being violent toward you or not, because if you don’t see it as wrong, you won’t recognize it.”

(P7)

Likewise, another relevant thematic axis was that of sexual consent, understood not as a one-time agreement but as a continuous and dynamic process. Participants emphasized the need to establish clear boundaries and to maintain ongoing communication that allows those boundaries to be reviewed and adjusted over time.

“I also think the issue of sexual consent within a relationship is important. […] You can say, ‘I’ve had a really good conversation with my partner, I’ve explained what I consent to and what I don’t,’ but these things can change. […] I change as a person, and so do you, so what you want and what I want can change.”

(P12)

On the other hand, the theme of changes in personal autonomy was identified as a sign of possible controlling dynamics. Participants reflected on how certain restrictions that did not previously exist, such as those related to clothing or social relationships, can indicate a progressive loss of freedom.

“If before I could go out wearing certain clothes that now I can’t, or if I used to talk to certain people and now I don’t, you have to ask yourself whether you’ve changed. But if now you feel controlled, or you feel there are things you can no longer do, that’s also a boundary.”

(P15)

At the same time, listening to one’s close social environment was highlighted as a strategy for identifying warning signs. Friends and individuals outside the relationship are perceived as key figures who can provide a more objective and protective perspective.

“I think it’s really important to listen to your friends and to people outside the relationship. From the outside, it’s very easy to see things, but from the inside, it’s very hard. […] If they tell you, ‘Be careful with this, it could be dangerous,’ it’s for a reason.”

(P18)

Another relevant aspect was the importance of prior information and training in order to identify signs of gender-based violence. Participants agreed that having prior knowledge of the characteristics of these forms of violence facilitates their detection, even in the early stages or in more subtle manifestations.

“If you know the signs or clues of what gender-based violence looks like, when it happens to you, you’ll probably recognize it right away. […] If you know what to look for, it’s easier than if it’s something completely unfamiliar to you. You just think it’s normal.”

(P1)

In situations of isolation or vulnerability, the need to make support resources visible (such as feminist organizations, associations, mutual aid groups, and psychological support services) was also emphasized. Participants noted that, in some cases, traditional support networks may not be available, whether due to geographical distance, migration, or controlling dynamics exerted by one’s partner.

“Sometimes, because of life circumstances, you don’t have that safe environment. […] These kinds of relationships don’t usually form overnight; over time, those behaviors start to develop. And by the time you realize it, you’re already deeply involved and don’t know how to get out.”

(P2)

In this context, the awareness of one’s own personal worth also emerged strongly as a turning point for establishing boundaries and breaking away from violent dynamics.

“I think that moment of reflection, ‘I don’t deserve this’, is important. You have to recognize what you’re not willing to allow and what you’re not willing to go through.”

(P2)

Finally, emotional validation was highlighted as an indicator of a healthy affective relationship. Participants noted that a partner who only responds positively when the other person is happy, but invalidates their negative emotions, may be demonstrating a form of conditional affection or even a dehumanizing idealization.

“If you say you’ve had a bad day and that person doesn’t share the feeling with you, but instead tells you that you’re overreacting, that’s a sign. […] That person doesn’t care for you; they idealize you. […] If that person can’t understand that I’m human, then I need to get out of there.”

(P10)

## 4. Discussion

This article explores the perceptions of university students in the Health Sciences regarding key dimensions for understanding and addressing gender-based violence in dating relationships. The results reveal a deep awareness of the various forms of violence present in affective relationships, as well as the central role that aesthetic pressure plays among the student profiles analyzed. Likewise, students’ preferences regarding the detection and approach to these forms of violence were identified.

In this context, romantic expectations, gender norms, and the idealization of one’s partner deeply shape affective relationships, influencing both their quality and the emotional experience of love. Students’ narratives reflect an idealized conception of romantic bonds, centered on the perfection of the other and the full satisfaction of one’s own emotional needs (Tuzgöl Dost & Aras, 2021). This idealization, together with the absence of critical reference points, fosters the gradual development of violent dynamics that are difficult to identify in their early stages. Consistent with previous research, the results of this study reveal a form of violence that evolves progressively, in which the initial manifestations, such as emotional manipulation, jealousy, or control, tend to be normalized (Castillo-Gonzáles et al., 2024).

Several studies (Nnubia & Eze, 2024) have indicated that conflicts in romantic relationships among university students intensify when difficulties arise in balancing academic responsibilities with quality time devoted to the relationship. The findings of this research reinforce this idea, showing how such imbalance can generate feelings of neglect and misunderstanding within the couple, which may ultimately lead to gender-based violence.

The normalization of sexist humor and its use as a justification for certain forms of violence also became evident. Because it is perceived as part of everyday life, it often goes unnoticed rather than being recognized as problematic. These results are consistent with studies warning that this type of humor reinforces gender stereotypes, conveys the idea of women’s inferiority, and contributes to the social acceptance of violence against women in university settings (Kanyemba & Naidu, 2022).

This normalization of discriminatory discourses and attitudes is closely linked to the presence of gender-based violence within romantic relationships, identified by participants as one of the most common expressions in dating contexts. Although less visible, behaviors such as manipulation, gaslighting, or emotional control have a profound impact on the mental and physical health of victims, as highlighted in previous studies (Lascorz et al., 2020; Parkinson et al., 2024).

In the same vein, psychological violence is confirmed as the most prevalent and one of the most harmful forms among university students (Tomaszewska & Schuster, 2021). The results of the present study identify a series of non-physical behaviors associated with this form of violence, such as control exerted by one’s partner (Mudayana et al., 2023), moral degradation through derogatory comments (Fernández-Antelo et al., 2020), emotional pain (Oflaz et al., 2023), indifference, lack of consideration (Esquivel-Santoveña et al., 2021), disrespect, and the absence of intimacy and individuality (Mudayana et al., 2023). Although often rendered invisible, these manifestations significantly affect the emotional health of those who experience them.

In addition, there is a growing presence of digital violence, repeatedly evidenced in students’ accounts. In particular, the phenomenon of digital coercive control was observed, in which technology is used to monitor, threaten, or exercise control over one’s partner. This form of abuse, characterized by its delocalized nature and constant availability, can intertwine with other forms of violence, as noted by various authors (Harris & Woodlock, 2019; Bosch & Gil-Juarez, 2021).

Another significant finding is the impact of aesthetic pressure and social comparison among peers, which reinforces normative and gendered aesthetic expectations, indirectly contributing to the perpetuation of gender-based violence in dating relationships (Meiksin et al., 2024). Individuals who feel pressured to conform to certain appearance standards are more likely to experience coercion or control within their relationships, as noted by Bianchi et al. (2021). In this context, the control of physical appearance emerges as a common strategy of domination, which may begin with seemingly harmless suggestions and progressively escalate into more explicit forms of control (Kwon & Park, 2019).

In response to these dynamics, the study highlights the importance of education in personal values and self-knowledge as key tools for setting boundaries and recognizing signs of emotional distress. This perspective aligns with pedagogical approaches that promote the transformation of beliefs about love and relationships, as well as the development of healthy norms in affective bonds. In this regard, both families and educational institutions play a fundamental role in preventing gender-based violence (Kwon & Park, 2019). The ability to identify symbolic or psychological forms of violence appears to be closely linked to processes of personal reflection and the development of critical awareness (Aprilianti & Yulindrasari, 2021; Willis & Jozkowski, 2019).

Within this same framework, the notion of sexual consent as a dynamic process represents a significant conceptual advancement over more static or contractual understandings of consent, as noted by Willis and Jozkowski (2019). Its presence in students’ discourses suggests a more critical and conscious understanding of affective dynamics.

Another noteworthy finding is the identification of changes in personal autonomy as indicators of controlling dynamics. This perception aligns with studies that emphasize the importance of observing transformations in everyday behavior as warning signs (Körner & Schütz, 2021). The progressive loss of freedom, particularly in aspects such as clothing or social relationships, is interpreted by participants as a form of violence that, although not always visible, has a strong emotional impact.

Closely related to the previous point, listening to one’s close social circle emerges as a complementary detection strategy, reinforcing the idea that dating violence should not be addressed solely from the individual sphere but also from a community-based and support network perspective. This relational dimension of care has been widely discussed in the literature on the prevention of gender-based violence (Lee & Wong, 2022; Pastor-Bravo et al., 2023).

Finally, emotional validation emerges as a key criterion for assessing the health of an affective relationship. A partner’s ability to provide emotional support and understanding is presented as an indicator of genuine affection, whereas its absence may be interpreted as a form of symbolic or emotional violence. This dimension, less explored in the existing literature, represents an original contribution of the present study by highlighting its relevance in the discourses of university students (Blázquez-Alonso et al., 2018).

The findings of this study have significant implications for Health Sciences students, both in their training and in their future professional practice. Indeed, health professionals play a key role in the prevention and early detection of gender-based violence victimization (Ameli et al., 2010; Baides Noriega, 2018). The development of a ten-point action plan is proposed for the detection and management of violence within the university setting, in collaboration with Equality Units, as well as the dissemination of results through accessible formats, such as video podcasts, to broaden their impact within the university community. These initiatives would help raise awareness among faculty and students, strengthening their capacity for identification, prevention, and action in the face of dating violence.

Among the limitations of this study, the methodological approach does not allow for the generalisation of the results. Nevertheless, the findings are consistent with existing literature and offer valuable insights into the experiences of young people facing situations of affective violence, as well as into the development of preventive strategies. Although the focus group technique does not permit an in-depth exploration of individual experiences, it is useful for capturing collective subjectivity, particularly considering that the participants are future health professionals. The inclusion of a mixed-gender group enabled the contrast of gender perspectives, thereby enriching the understanding of relational dynamics. However, this technique may also result in unequal levels of participation or the omission of experiences and viewpoints that could have further expanded the collective narratives. Bearing this in mind, the research team implemented measures to minimise this potential bias—such as creating a safe environment, using open-ended questions, and emphasising confidentiality—although we recognise that it cannot be completely eliminated. Furthermore, some of the themes that emerged—particularly those related to digital violence—may have been influenced, at least in part, by the researchers’ prior areas of interest. Although an inductive approach was followed and efforts were made to minimise interference through independent coding and consensus-based interpretation, researcher reflexivity remains a crucial element. In this regard, we consider that future studies should incorporate additional strategies, such as triangulation with external researchers, to further mitigate this potential influence. Additionally, however recruitment and participation took place outside regular class hours to avoid any perception of coercion and ensure voluntary participation, this strategy may also have influenced who ultimately chose to take part and, therefore, the type of knowledge generated.

Moreover, the mixed-gender composition of the group, together with the gender of the moderators, may have influenced interactional dynamics and shaped what participants felt comfortable sharing, potentially introducing social-desirability effects. Gender was also addressed in cis-binary terms, limiting the study’s ability to capture the perspectives of gender-diverse students.

Additionally, the homogeneity of the sample must be acknowledged as a relevant limitation. All participants identified as Caucasian, and no information was collected regarding sexual orientation, relationship structure, or migration background. This lack of diversity restricts the range of perspectives represented and limits the study’s ability to examine how experiences and perceptions of dating violence may intersect with racialised identities, minority stress, or migration trajectories. Moreover, some discussions—such as those concerning same-sex relationships—reflected perceptions or vicarious understandings rather than necessarily lived experience. The section addressing racism also revealed potentially racialised assumptions that should be interpreted within existing structural power dynamics. These considerations highlight the need for future studies to include more diverse samples and to adopt an explicitly reflexive approach when analysing narratives related to cultural and identity differences.

The group format may also have introduced social desirability bias, potentially limiting the spontaneity and authenticity of participants’ contributions. This risk may have been reinforced by the fact that three of the ten participants were previously acquainted with members of the research team, including one who had a teaching relationship with the students. Although this individual did not moderate the session and the independence of the study was emphasised, we acknowledge that this dynamic may have shaped participants’ responses. For this reason, we suggest that future research should avoid involving students with direct teaching links to the research team or incorporate moderators who are entirely external to the academic context.

Finally, it is considered a priority for future research to analyse the impact of co-created interventions between students and health professionals, in order to evaluate their effectiveness in both university and healthcare settings.

## 5. Conclusions

The analysis of the narratives of university students in the Health Sciences has made it possible to identify key dimensions for understanding and addressing gender-based violence in dating relationships from an experiential perspective. The testimonies reveal that the detection of such forms of violence is not always immediate, especially when they manifest subtly or progressively. In this context, self-knowledge, education in values, emotional validation, and relational awareness with clear boundaries emerge as fundamental pillars for the development of healthy affective relationships.

Likewise, the significant presence of psychological and digital violence is highlighted, underscoring the need to broaden the focus beyond physical or explicit forms. These manifestations, often rendered invisible, directly affect the autonomy, self-esteem, and decision-making capacity of those who experience them.

The importance of having support networks, both personal and institutional, emerges as a key strategy for contrasting perceptions, receiving accompaniment, and accessing resources in situations of vulnerability. In this regard, active listening within one’s environment, the visibility of specialized services, and prior training in gender-based violence are configured as highly valuable preventive tools.

As key educational spaces, universities face the challenge of creating safe environments that foster support and make visible strategies for the prevention, care, and reparation of gender-based violence. These should be spaces where students can reflect, express themselves, learn, and access support resources effectively. Ultimately, this study contributes to enriching the understanding of dating violence and strengthening detection and response strategies from a transformative perspective.

## Figures and Tables

**Table 1:** Participants.

Participant	Age	Gender	Year of Study	Health Sciences Studies	Ethnicity
P1	21	Female	First	Nursing studies	Caucasian
P2	21	Female	First	Nursing studies	Caucasian
P3	20	Male	First	Nursing studies	Caucasian
P4	21	Female	First	Nursing studies	Caucasian
P5	23	Male	Second	Medicine studies	Caucasian
P6	21	Female	Second	Medicine studies	Caucasian
P7	27	Female	First	Nursing studies	Caucasian
P8	21	Male	Second	Medicine studies	Caucasian
P9	21	Female	Second	Medicine studies	Caucasian
P10	19	Female	First	Nursing studies	Caucasian

**Table 2:** Research Topics and Theme Description.

Theme	Subtheme	Theme Description
Manifestations of dating Violence	Characteristics of Dating Violence	Identify and analyze patterns of abusive behaviors—physical, emotional, psychological, or sexual—used by one partner to exert power and control within romantic relationships.
	Types of violence	Identify and classify the different forms of abuse present in dating relationships, including physical, emotional, psychological, and sexual violence.
	Aesthetic pressure in Dating	Explore and categorize the influence of societal beauty standards on individuals within romantic relationships, and how these expectations affect self-perception and partner dynamics.
Strategic Approaches and Recommendations for the Prevention and Intervention of Dating Violence	Education and prevention	Examine educational strategies and preventive measures aimed at raising awareness, promoting healthy relationships, and reducing the incidence of dating violence among young people.

**Table 3:** Overview of Main Categories and Subcategories Ranked by Frequency.

Main Category	Subcategories
Psychological violence	Loss of intimacy
	Emotional pain
	Emotional dependency
	Control Degradation
	Emotionally Manipulate
	Derogatory comments
	Lack of respect
	Ignoring or not taking their opinions seriously
Dating Violence	Expectations in a Relationship
	Romantic Relationships
	Types of Violence
	Student Context
	Relational and Social Conflict
	Perception of Violence
Digital violence	Violation of trust
	Digital control
	Influence of social media
Subtle Violence	Unnoticed violence
	Non-obvious violence
	Subtle behaviors
	Subliminal violence
	Double-meaning messages
	Micro violence
Aesthetic pressure	Social comparison
	Appearance control
	Aesthetic expectations
	Aesthetic violence
Education and Prevention	Setting Boundaries
	Emotional Support
	Intervention and Help
	Self-awareness
	Assertive Communication
	Relational Awareness
	Inclusion and Diversity
	Educational and Preventive Measures
	Critical Thinking
	Gender Equality
Racism and Discrimination	LGBT phobia
	Patriarchal System
	Personal Values
Verbal violence	Violent acts
Sexual violence	
Physical violence	

## Data Availability

Due to ethical restrictions, the data supporting the findings of this study cannot be made publicly available.

## Supplementary Materials

The following supporting information can be downloaded at https://www.mdpi.com/article/10.3390/bs16050630/s1, Table S1: The Consolidated Criteria for Reporting Qualitative Research (COREQ) checklist for the reporting of qualitative results.
