# Supplementary material for: New Values, New Lives, and Emerging Dating Violence: Insights on Detection and Intervention from Health Sciences Students
Source: Behav Sci (Basel). 2026 Apr 23;16(5):630. doi: 10.3390/bs16050630 (PMC13203934; doi:10.3390/bs16050630)
Supplement: Supplementary file 1 [file behavsci-16-00630-s001.zip › behavsci-4224473-supplementary.pdf]

Supplementary Materials

Table S1. Consolidated criteria for reporting qualitative studies (COREQ): 32-item checklist

No	Item	Guide questions/description	Response	Checklist
**Domain 1: Research team and reflexivity**<br>Personal Characteristics				
1.	Interviewer/ facilitator	Which author/ s conducted the interview or focus group? Invest	Principal Investigator	pg.1
2.	Credentials	What were the researcher's credentials? E.g. PhD, MD	PhD	pg.1
3.	Occupation	What was their occupation at the time of the study?	Assistant Professor of Psychological Sciences	pg.1
4.	Gender	Was the researcher male or female?	Female	pg.1
5.	Experience and training	What experience or training did the researcher have Relationship with participants	10 years of research experience. Some participants had previously attended the course on Psychosocial Sciences.	pg. 6
Relationship with participants				
6.	Relationship established	Was a relationship established prior to study comm	Only three participants had attended classes taught by the Principal Investigator.	pg. 6
7.	Participant knowledge of the Interviewer	What did the participants know about the researcher? eg interviewer research	At the beginning of the study, they were only aware of the topic to be explored in the research.	pg. 4
8.	Interviewer characteristics	What characteristics were reported about reasons and interests in the research topic	The research team reported that their interest in the topic stemmed from prior clinical experience with gender-based violence cases and a commitment to improving detection and care in nursing education. One of the research team members is the Vice-Rector for Equality at the university, which means the results of this study have implications beyond the field of nursing.	pg. 6
**Domain 2: study design**<br>Theoretical framework				
9.	Methodological orientation and Theory	What methodological orientation was stated to underpin the study? e.g. grounded theory, discourse analysis,	Phenomenology	pg. 4

		ethnography, phenomenology, content analysis		
**Participant selection**				
10.	Sampling	How were participants selected? convenience, consecutive, snowball	Convenience sampling	pg. 5
11.	Method of approach	How were participants approached? eg. face-to face, telephone, mail,	Focus grup face to face	pg. 5
12.	Sample size	How many participants were in the study?	10 participants	pg. 5
13.	Non-participation Setting	How many people refused to participate or dropped out? Reasons?	One person seemed interested in participating but did not attend the session and did not notify the team. Two people didn't sign the consent form.	pg. 5
**Setting**				
14.	Setting of data collection	Where was the data collected?	The data were collected in a university classroom setting during scheduled sessions of the nursing/medicine program.	pg. 4
15.	Presence of non-participants	Was anyone else presen besides the participants and researchers?	No individuals other than the participants and the research team were present during the focus group.	pg. 4
16.	Description of sample	What are the important characteristics of the sample?	The sample consisted of 10 undergraduate nursing students (ages 19–23), enrolled in the first and second year of a health sciences program, with prior exposure to psychosocial health topics.	pg. 5
**Data collection**				
17.	Interview guide	Were questions, prompts, guides provided by the authors? Was it pilot tested?	Yes, a semi-structured interview guide with open-ended questions and prompts was provided to ensure consistency of the focus group. No pilot testing was conducted; The interview guide was developed based on previous research and adapted from guidelines provided by governmental source	pg. 6
18.	Repeat interviews	Were repeat interviews carried out? If yes, how many?	No repeat focal grups were conducted; each participant attended one focus group session	pg. 5
19.	Audio/visual recording	Did the research use audio or visual recording to collect the data?	Audio recordings were used for the focus group to ensure accurate transcription.	pg. 6
20.	Field notes	Were field notes made during and/or after the interview or focus group?	Field notes were taken during and immediately after each focus group to capture non-verbal cues and contextual details.	pg. 6
21.	Duration	What was the duration of the interviews or focus group?	It takes 2 hours	pg. 6

22.	Data saturation	Was data saturation discussed?	Data saturation was considered achieved in the first focus group, as no new relevant themes emerged in the subsequent sessions. Therefore, it was not necessary to repeat interviews or add additional groups.	pg. 6
23.	Transcripts returned	Were transcripts returned to participants for comment and/or correction?	Transcripts were returned to participants for comment or correction	pg. 7
**Domain 3: analysis and findings**<br>Data analysis				
24.	Number of data coders	How many data coders coded the data?	Two independent researchers coded the data to ensure reliability	pg. 6
25.	Description of the coding tree	Did authors provide a description of the coding tree?	Yes, a detailed coding tree was developed and included in the main text.	pg. 8
26.	Derivation of themes	Were themes identified in advance or derived from the data	Themes were developed through a combination of inductive and deductive approaches, applying an abductive reasoning process within a thematic analysis framework.	pg. 7
27.	Software	What software, if applicable, wns used to manage the data?	We used Atlas.ti	Pg. 7
28.	Participant checking	Did participants provide feedback on the findings	The participants provided feedback on the findings.	Pg. 7

Tong, A., Sainsbury, P., & Craig, J. (2007). Consolidated criteria for reporting qualitative research (COREQ): A 32-item checklist for interviews and focus groups. International Journal for Quality in Health Care, 19(6), 349–357. https://doi.org/10.1093/intqhc/mzm042
